# Emergency department utilisation among older adults—Protocol for a systematic review of determinants and conceptual frameworks

**DOI:** 10.1371/journal.pone.0265423

**Published:** 2022-06-03

**Authors:** Xuan Rong Tang, Faraz Zarisfi, Pin Pin Pek, Fahad Javaid Siddiqui, Rahul Malhotra, Yu Heng Kwan, Tiah Ling, Andrew Fu Wah Ho, Marcus Eng Hock Ong

**Affiliations:** 1 Yong Loo Lin School of Medicine National University of Singapore, Singapore, Singapore; 2 Department of Emergency Medicine, Singapore General Hospital, Singapore, Singapore; 3 Prehospital and Emergency Research Centre Duke-NUS Medical School, Singapore, Singapore; 4 Duke-NUS Medical School, Program in Health Services and Systems Research, Singapore, Singapore; 5 Accident and Emergency Department, Changi General Hospital, Singapore, Singapore; University of Copenhagen: Kobenhavns Universitet, DENMARK

## Abstract

**Background:**

Older adults aged 65 years and above have a disproportionately higher utilization of emergency healthcare, of which Emergency Department (ED) visits are a key component. They experience higher degree of multimorbidity and mobility issues compared to younger patients, and are consequently more likely to experience a health event which requires an ED visit. During their visit, older adults tend to require more extensive workup, therefore spending a greater amount of time in the ED. Compared to the younger population, older adults are more susceptible to adverse events following discharge. Considering these factors, investigating the determinants of ED utilisation would be valuable. In this paper, we present a protocol for a systematic review of the determinants of ED utilisation among communitydwelling older adults aged 65 years and above, applying Andersen and Newman’s model of healthcare utilisation. Furthermore, we aim to present other conceptual frameworks for healthcare utilisation and propose a holistic approach for understanding the determinants of ED utilisation by older persons.

**Methods:**

The protocol is developed in accordance with the standards of Campbell Collaboration guidelines for systematic reviews, with reference to the Cochrane Handbook for Systematic Review of Interventions. Medline, Embase and Scopus will be searched for studies published from 2000 to 2020. Studies evaluating more than one determinant for ED utilisation among older adults aged 65 years and above will be included. Search process and selection of studies will be presented in a PRISMA flow chart. Statistically significant (p < 0.05) determinants of ED utilisation will be grouped according to individual and societal determinants. Quality of the studies will be assessed using Newcastle Ottawa Scale (NOS).

**Discussion:**

In Andersen and Newman’s model, individual determinants include predisposing factors, enabling and illness factors, and societal determinants include technology and social norms. Additional conceptual frameworks for healthcare utilisation include Health Belief Model, Social Determinants of Health and Big Five personality traits. By incorporating the concepts of these models, we hope to develop a holistic approach of conceptualizing the factors that influence ED utilisation among older people.

**Systematic review registration:**

This protocol is registered on 8 May 2021 with PROSPERO’s International Prospective Register of Systematic Reviews (CRD42021253770).

## 1 Background

The emergency department (ED) acts as a bridge between the community and hospital, where people are referred by their primary care physicians (PCPs) or by themselves, and plays a crucial role in regulating hospital admissions [1]. In the healthcare system, the ED is vital in supporting primary care by caring for patients outside of office hours or performing advanced diagnostic investigations [1]. In recent years, there has been a surge in ED visits [2, 3], as seen in the United States where the number of visits per 1000 people have increased from 369 to 458 visits between 1995 to 2016 [4]. Similarly in Singapore, the number of ED visits have risen by 250,000 from 2007 to 2013 [5]. Adults aged 75 years and above had the second highest visit rate of 52 visits per 100 people [6]. The number of older persons, aged 65 years and above, is expected to rise [7], as is the frequency of ED utilisation by this population sub-group [4].

Older adults contribute a disproportionate number of visits to the ED [8, 9] and tend to require more extensive workup, therefore spending a greater amount of time in the ED [4]. Furthermore, there is higher resource expenditure among this population in the form of advanced investigations such as computed tomography and magnetic resonance imaging [2]. Compared to their younger counterparts, they are at higher risk of hospitalisation as well as adverse events when they visit the ED [10]. In the United States, expenses incurred from inpatient care accounts for 31% of national healthcare spending [1]. In addition, they have an increased susceptibility to hospital acquired pneumonia [11]. Older adults who were discharged from the ED had a reduction in their mobility within the community, which may not improve within a year from discharge [12].

Generally, older adults have multiple comorbidities and complex medical issues that may require care beyond the PCP level [5]. In a systematic review done by [5] adapting Andersen’s Behavioural model to study the determinants of ED utilisation [5], need or illness factors were shown to be a significant determinant across many studies. This signifies that older adults truly require emergent care and may be too acutely ill to await an appointment at the PCP. In certain situations, the process of deterioration could have been deterred with regular follow-up care with PCPs [13]. Moreover, McCusker found that predisposing and enabling factors that increase use of PCP will lead to a decrease in ED utilisation [5]. Indeed, the presence of barriers to primary care was identified in another study as one of the reasons why older adults turn to the ED in desperation to resolve their issues. Some were told by the PCP staff to visit the ED if they felt it was urgent [14]. The influence of predisposing and enabling factors seen shows there exists a multitude of factors that should be explored as well. This is visualised through the model proposed by Andersen and Newman [15], where healthcare utilisation is determined by societal or individual factors.

Individual determinants include predisposing, enabling and illness factors. Predisposing factors are patient sociodemographic characteristics that can incline or deter a patient from utilising healthcare. For instance, older adults with lower education level were found to have an increase in ED utilisation [16]. This could be explained by the associated lower health literacy, which leads to higher number of ED visits [17]. Enabling factors encompass the influence of family and community, with examples including marital status, living conditions and geographical accessibility to PCPs or EDs. Older adults residing in rural areas had a lower ED utilisation rate than those in urban areas, as they may reside further from healthcare facilities [18]. Need or illness factors can be divided into perceived (subjective) need or evaluated (objective) need [19, 20]. Older adults with comorbidities are predisposed to complications of their chronic illnesses and could have a lower threshold to visit the ED.

Societal determinants include technology and norms. Technology will help promote the efficacy of physicians providing care within the healthcare system, which can influence the decision of the population to seek medical care [15]. An example of this would be the availability of X-rays and blood investigations at the ED which may not be available at the PCP level [21]. Societal norms arise from governmental policies as well as societal values and beliefs [15]. For instance, the stigma associated with mental health issues impedes help-seeking behaviour among people who need them and potentially deters them from utilising healthcare [22]. Health insurance policies and medical subsidies by the government play a key role in a person’s decision to utilise healthcare resources [15], as demonstrated in Anderson’s study where healthcare utilisation was lower among people without insurance coverage [23].

The stress on the ED needs to be addressed to avoid jeopardising the quality of care provided and slow the surge in healthcare expenditure [3]. The ill effects on older adults outlined above with regards to ED visitation emphasises the need to investigate the determinants of ED utilisation. With identification of these factors, we may be able to mitigate the number of visits to the ED by the older adult population through primary, secondary and tertiary prevention. Hence, in this paper, we outline the protocol for a systematic review of the determinants of ED utilisation among older persons (aged 65 years and above), using the framework proposed by Andersen and Newman [15].

In addition to Andersen and Newman’s model, other frameworks have been used to explain health services utilization. These include the Health Belief Model [24, 25], Social Determinants of Health [26] as well as Big Five personality traits [27, 28]. Lutz et al. devised a framework to understand ED utilisation by describing the factors that influence the decision of visiting the ED or primary care [29]. In addition, He et al. proposed a modified Andersen and Newman’s model to visualise ED utilisation [30]. By incorporating concepts of all the models utilised in our review, we hope to develop a more holistic approach of conceptualizing the factors that influence the decision of older adults to visit the ED.

Our protocol was developed in accordance with the standards of Campbell Collaboration guidelines for systematic reviews [31], with reference to the Cochrane Handbook for Systematic Review of Interventions [32]. This protocol is registered with PROSPERO’s International Prospective Register of Systematic Reviews (CRD42021253770).

## 2 Methods

### 2.1 Objectives

The primary aim of our study is to review the existing literature in the area of ED utilisation among older adults (aged 65 and above), under the headings of individual and societal determinants to better understand the reasons for the disproportionate ED usage among older adults.

The secondary aim is to present other conceptual frameworks that have been used for explaining healthcare utilisation, and propose a more holistic approach of conceptualizing the factors that influence the decision of older persons to visit the ED.

### 2.2 Electronic searches

PubMed, Embase and Scopus will be searched. Additional papers would be identified through handsearching. Grey literature will be searched in OpenGrey. Search will be limited from 2000 to 2020 and papers in the English language only.

The search strategy was developed in collaboration with a university librarian. Medical Subject Headings (MeSH) terms that will be used are “aged”, “health services for the aged”, “health services accessibility”, “health care surveys”, “emergency service, hospital” and “emergency medicine”. Emtree subject headings that will be used are “aged”, “hospital”, “emergency health service”, “emergency medicine”, “emergency ward”. Full list of keywords with boolean terms used are available in the appendix.

### 2.3 Inclusion criteria

Studies that evaluate one or more determinants for ED utilisation among community-dwelling older persons aged 65 and above will be included. The reasons for presentation to a hospital ED, the frequency of utilisation or time spent in the ED must be measured in the studies. Papers that are published in English language between 2000 to 2020 will be considered.

### 2.4 Exclusion criteria

Studies that evaluate determinants for healthcare utilisation in other contexts such as urgent care centres or primary care clinics that are open beyond office hours will be excluded. Papers that merely study presenting complaints of older patients, or studies evaluating determinants of revisits or frequent visits will be excluded.

### 2.5 Selection of studies

**Fig 1:**
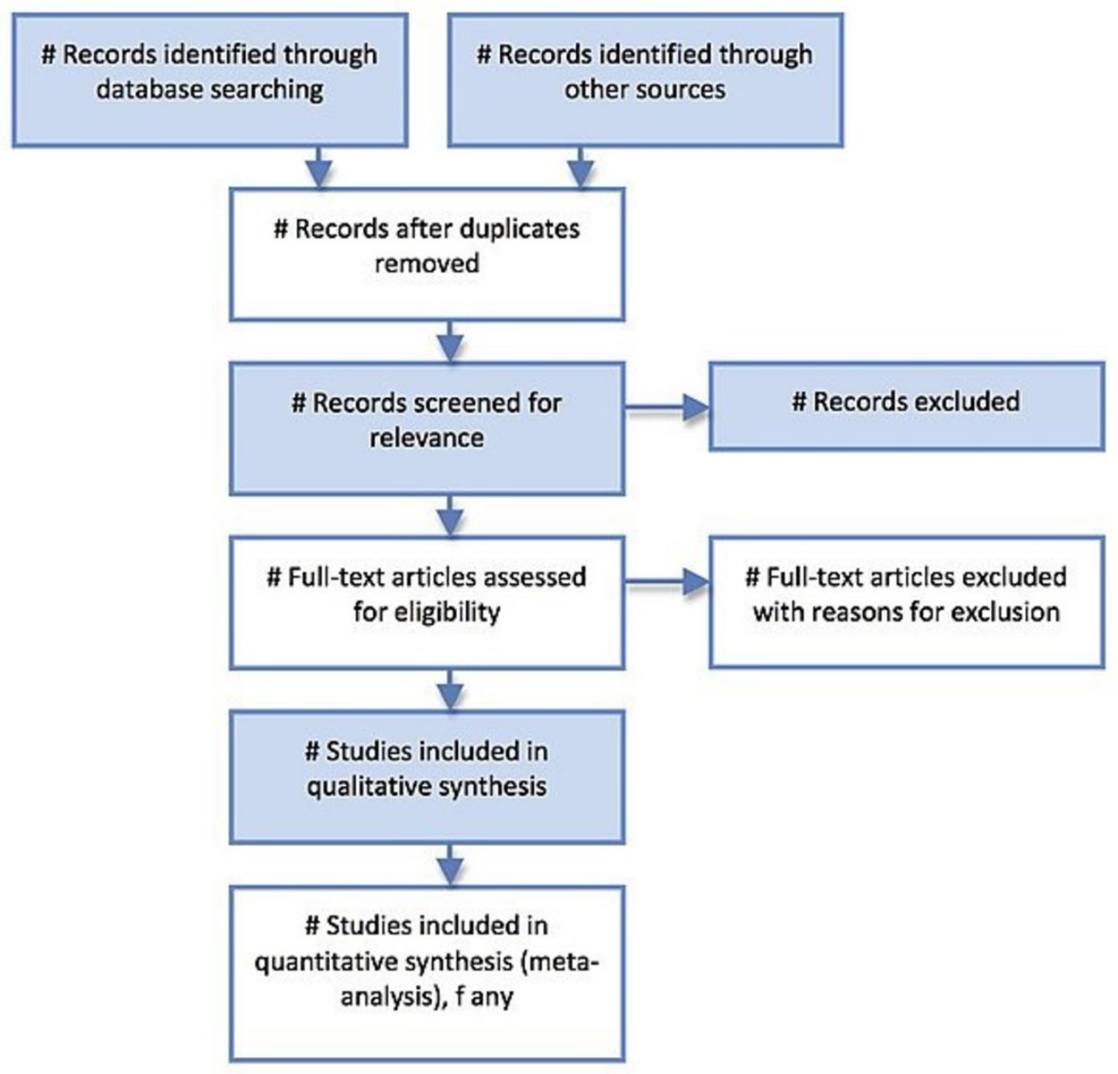
Sample PRISMA flow chart.

The search and selection process will be displayed in a Preferred Reporting Items for Systematic Review and Meta-analysis (PRISMA) flow chart [33], as shown in Fig 1.

At least two independent reviewers will conduct the search and selection of studies for each of the databases. A similar search strategy will be applied to each database and citations exported to EndNote X9. Papers identified from references and grey literature will also be included. Duplicates will be removed. Title and abstracts of the search results will then be screened independently for eligibility by two reviewers. Upon screening and shortlisting eligible papers based on the title and abstract, the full text of these articles would be obtained and assessed further for suitability to include in the systematic review. Articles that are excluded for various reasons will be taken note of, in which the justification will be presented in the flow chart and in the manuscript. In the event of any conflict between two reviewers, the opinion of a third, senior reviewer would be consulted.

### 2.6 Data extraction and management

Training will be provided to reviewers through the Campbell Collaboration website training page, which comprises resources on conducting a systematic review [34]. At least two reviewers will be required for data extraction for each article and any discrepancies resolved through discussion and consensus with a third reviewer. The following study characteristics will be identified and extracted from the eligible studies: (1) Author and year, (2) country, (3) study design, (4) study population, (5) sample size and sampling methods, (6) outcome variable(s), (7) data source for outcome(s), (8) determinants examined, (9) data source for determinants. In this table (Table 1), the determinants examined will be classified according to societal determinants (technology and norms) and individual determinants (predisposing, enabling and illness factors). The statistically significant determinants of ED utilisation identified in the studies, together with its effect size, will be presented in the next table (Table 2).

**Table 1:** Characteristics of included studies (dummy).

Author (year)	Country	Design of study	Study population	Sample size and sampling methods	Outcome variable	Data source for outcome	Determinants examined		Data source for determinants
							Individual determinants	Societal determinants	
									

**Table 2:** Statistically significant determinants of ED utilisation based on multivariable analysis (dummy table).

Author (year)	Individual determinants			Societal determinants		Other significant factors & effect size
	Predisposing factors and effect size	Enabling factors and effect size	Illness level and effect size	Technology and effect size	Norms and effect size	
						

## 3 Data analysis

The study design and data sources of each study will be presented under the characteristics of included studies in Table 1, together with the list of determinants examined by the various studies. This will provide an overview of the determinants of ED utilisation among patients aged ≥ 65 years.

Review Manager (RevMan) 5 will be employed for this systematic review. The determinants that are significantly associated with ED utilisation will be identified, through either univariable or multivariable analysis conducted in the included studies. Statistically significant determinants of ED utilisation (p < 0.05) will be identified and presented in Table 2. The effect size of the statistically significant determinants will be pooled, if possible, for meta-analysis. Qualitative data will be included for systematic review. Adopting the Andersen and Newman model, the determinants will be classified according to individual-level determinants (and therein as illness level, predisposing factors and enabling factors) and societal-level determinants (and therein technology and norms). Determinants that do not fall under this classification will be listed under “other significant factors”—their identification will be useful in informing and proposing modifications to Andersen and Newman’s model. If deemed appropriate, we will then devise a modification of the model that will be applicable in the context of ED utilisation.

### 3.1 Criteria for assessing the quality of the qualitative evidence

The Newcastle Ottawa Scale (NOS) will be used for evaluation of the quality of included studies, which will comprise mainly non-randomised studies [35]. The criteria for evaluation in NOS and reliability between raters have been validated [36]. As the original NOS only includes assessment of quality of cohort and case-control studies [37], a modified version of the scale would be adopted for cross-sectional studies [38]. This modified NOS has been used by a prior study [39] with similar criteria of assessment—selection, comparability and outcomes of study. The appraisal of the studies will be presented in Table 3.

**Table 3:** Quality assessment of included studies using NOS (dummy table).

Author (year)	Selection	Comparability	Outcome
			

### 3.2 Dealing with missing data

In the event of missing information (such as effect sizes of statistically significant determinants), the corresponding authors of the relevant studies would be contacted to obtain the missing data.

## 4 Discussion

Using Andersen and Newman’s model of healthcare utilisation, there is a comprehensive viewpoint on the determinants of ED utilisation which takes into account geographical distance and variations in healthcare systems across different societies. According to the World Health Organisation, older persons are aged 65 years and above, hence justifying our cut-off age [40]. Even though our review includes only older patients aged 65 and above, some of the determinants (such as geographical distance) could also be applicable to younger age groups as they are not exclusive to the older adult population. Therefore, our study findings have the potential to be generalised across other populations.

One limitation of our study is the exclusion of non-English papers, which could limit the generalisability of our findings to non-English speaking countries.

Ethics approval is not required as this is a systematic review. We intend to submit the completed review for peer-reviewed publication and to present our findings at relevant meetings and conferences.

### 4.1 Preliminary timeframe

Table 4 below presents the estimated duration required for each stage of the systematic review. In total, the review would take an estimated 6 months to complete.

**Table 4:** Estimated duration required for each stage of the systematic review.

Stage of review	Duration (weeks)
Training and pilot testing on the inclusion criteria	3
Search for eligible results	4
Screening results from the literature search	3
Training and pilot testing the study coding procedure	3
Extraction and analysis of data from eligible research reports	4
Preparation of the final review report	8

### 4.2 Plans for updating the review

No plans are made to update the systematic review at the time of writing. In the event of any changes to the original protocol, they will be presented in the published review.

## Supporting information

S1 Checklist PRISMA-P 2015 checklist. (DOCX)

S1 File (PDF)

S1 Text (PDF)
